# Low-Dose Propranolol Prevents Functional Decline in Catecholamine-Induced Acute Heart Failure in Rats

**DOI:** 10.3390/toxics10050238

**Published:** 2022-05-07

**Authors:** Cheng-Ken Tsai, Bo-Hau Chen, Hsin-Hung Chen, Rebecca Jen-Ling Hsieh, Jui-Chen Lee, Yi-Ting Chu, Wen-Hsien Lu

**Affiliations:** 1 Department of Cardiovascular Surgery, Zuoying Branch of Kaohsiung Armed Forces General Hospital, Kaohsiung 81342, Taiwan; m871373@gmail.com; 2 Department of Pediatrics, Taoyuan Armed Forces General Hospital, Taoyuan 32551, Taiwan; dreamvenice@hotmail.com; 3 Department of Medical Education and Research, Kaohsiung Veterans General Hospital, Kaohsiung 813414, Taiwan; shchen0910@gmail.com (H.-H.C.); rjhsieh500@gm.ym.edu.tw (R.J.-L.H.); 4 School of Medicine, National Yang-Ming University, Taipei 112, Taiwan; 5 Department of Pediatrics, Kaohsiung Veterans General Hospital, Kaohsiung 813414, Taiwan; xu3bjo45p@gmail.com (J.-C.L.); ytchuchu@vghks.gov.tw (Y.-T.C.); 6 Institute of Biomedical Sciences, National Sun Yat-sen University, Kaohsiung 804, Taiwan

**Keywords:** beta-blocker, catecholamine, heart failure, myocardial dysfunction, propranolol

## Abstract

Severe hyper-catecholaminergic states likely cause heart failure and cardiac fibrosis. While previous studies demonstrated the effects of beta-blockade in experimental models of single-catecholamine excess states, the detailed benefits of beta-blockade in more realistic models of hyper-adrenergic states are less clearly understood. In this study, we examined different therapeutic dosages and the effects of propranolol in rats with hyper-acute catecholamine-induced heart failure, and subsequent cardiopulmonary changes. Rats (n = 41) underwent a 6 h infusion of epinephrine and norepinephrine alone, with additional low-dose (1 mg/kg) or high-dose propranolol (10 mg/kg) at hour 1. Cardiac and pulmonary tissues were examined after 6 h. Catecholamine-only groups had the lowest survival rate. Higher doses of propranolol (15 mg/kg) caused similarly low survival rates and were not further analyzed. All low-dose propranolol rats survived, with a modest survival improvement in the high-dose propranolol groups. Left ventricular (LV) systolic pressure and LV end-diastolic pressure improved maximally with low-dose propranolol. Cardiac immunohistochemistry revealed an LV upregulation of FGF-23 in the catecholamine groups, and this improved in low-dose propranolol groups. These results suggest catecholamine-induced heart failure initiates early pre-fibrotic pathways through FGF-23 upregulation. Low-dose propranolol exerted cardio-preventative effects through FGF-23 downregulation and hemodynamic-parameter improvement in our model of hyper-acute catecholamine-induced heart failure.

## 1. Introduction

Catecholamines are an important mediator during physiological stress. Several critical conditions can cause the elevation of serum catecholamines, from endogenous secretions in the setting of septic shock [1] to exogenous administrations during acute resuscitation. Excessive serum catecholamines may likewise induce heart failure [2]. In animal models of hyperadrenergic states, most models were constructed through single catecholamine use, with a focus upon long-term effects. Effects of chronic exposure to epinephrine (E) include biventricular heart failure and ventricular remodeling [3], while rats infused with continuous norepinephrine (NE) developed left ventricular hypertrophy [4]. Fibroblast growth factor 23 (FGF-23), a novel inducer of cardiac hypertrophy and fibrosis through pro-fibrotic gene transcription, is speculated to participate in cardiac remodeling with possible reversibility [5,6]. 

Although the individual effects of E and NE have been studied in various animals, in vivo models investigating the combined effects of E and NE co-administration upon cardiopulmonary physiology are relatively scarce, even though they are more representative of clinical hyperadrenergic states, including sepsis, chronic heart failure, or the iatrogenic supratherapeutic administration of catecholamines [1,3,7]. On the other hand, prospective analyses of patients in shock provide insight into the real-world effects of combined catecholamines. In cohorts with single E use, higher mortality rates with complications including arrhythmia, lactate acidosis, and cardiac stress were observed than in those with additional NE [8]. A recent meta-analysis examined the clinical use of beta-blockers in patients with sepsis and septic shock, with results revealing an improvement in mortality in 4 out of 6 studies [9]. However, detailed mechanisms of these pathways are still lacking, with few real-world applications of beta-blockade in these scenarios. With more information and mechanistic explanations from the bench-side, clinical utilization may become more common. 

We previously demonstrated, in our preceding work, that rat models injected with E and NE displayed significant cardiopulmonary impairment and biventricular dysfunction compared with catecholamine monotherapy, in accordance with previous studies [10]. Therefore, we sought to further investigate the consequences and methods of mitigating hyperadrenergic states through an experimental model of greater clinical relevance. Although beta-blockade is commonly known to be beneficial in experimental models of single catecholaminergic states, the mechanisms and benefits of beta-blockade in realistic hyperacute adrenergic states through combined excess E and NE administration are less explored. We hereby propose a catecholamine-induced acute heart failure model in rats through combined catecholamines, with the aim of alleviating hemodynamic dysregulation through propranolol. We hypothesize that non-selective beta-blockade, through the downregulation of beta-adrenergic receptors in catecholamine excess, improves hemodynamic parameters and pre-fibrotic markers such as FGF-23 [11]. 

## 2. Materials and Methods

### 2.1. Experimental Animals

All animal research protocols were approved by the Institutional Animal Care and Use Committee of Kaohsiung Veterans General Hospital (Identification code: 2020-A012, 2021-A002, and 2022-A011; date of approval: 17 May 2019, 11 March 2020, and 29 March 2021). All adult male Sprague Dawley rats (8 to 10 weeks old, 320~380 g, n = 41) were purchased from BioLASCO Taiwan Co., Ltd. (Taipei, Taiwan), and randomly assigned into the following five groups: (1) sham (0.9% saline infusion, n = 6), (2) E + NE (E and NE infusion, n = 10), (3) E + NE treated with low-dose propranolol (E and NE infusion with propranolol 1 mg/kg, n = 7), (4) E + NE treated with high-dose propranolol (E and NE infusion with propranolol 10 mg/kg, n = 8), (5) E + NE treated with higher-dose propranolol (E and NE infusion with propranolol 15 mg/kg, n = 10).

Animals were anesthetized with intraperitoneal (1 g/kg) urethane. Left femoral veins were cannulated with a syringe pump for E (Taiwan Biotech Co., Ltd., Taoyuan, Taiwan) and NE (Tai Yu Chemical & Pharmaceutical Co., Ltd., Hsinchu, Taiwan) infusion at a steady rate (4.5 μg/kg/min and 6.8 μg/kg/min, respectively) for 6 h during the experiment. Right femoral veins were cannulated for propranolol injection. Propranolol (# P0884, Sigma-Aldrich Co., St. Louis, MO, USA) was dissolved in dd-H20 (pH3.0) and diluted with 0.9% saline (YF Chemical Corp., New Taipei, Taiwan) to different concentrations (1, 10, or 15 mg/kg) beforehand. After 1 h of continuous E and NE infusion, propranolol was injected once into rats. 

### 2.2. Hemodynamic Data Acquisition

Commercial pressure catheters SPR-513 and SPR-407 were connected to PCU-2000 control units (Millar Inc., Houston, TX, USA) and the PowerLab 35 Series data-acquisition system with LabChart Pro and analyzed with a blood pressure analysis program (ADInstrument Inc., Colorado Springs, CO, USA). The SPR-513 catheter was inserted through the right jugular vein into the right ventricle (RV) and confirmed by the typical RV pressure curve. The SPR-407 catheter was placed in the right carotid artery and advanced into the left ventricle (LV). Biventricular systolic and end-diastolic pressures, heart rates, and other hemodynamic parameters were recorded. The contractility index was calculated according to the formula provided by the PowerLab 35 acquisition system. It is calculated as follows: dP/d*t*_max_ divided by the pressure (*p*) at the time of max dP/d*t*_max_, where max dP/d*t* is defined as the steepest slope during the downstroke of the pressure curve.

### 2.3. Serum Analysis of Cardiac Markers

Six hours post-catecholamine infusion, blood from the rats’ heart were collected into MiniCollect tubes (MiniCollect^®^ Z Serum Sep, Greiner Bio-One GmbH, Kremsmünster, Austria) and centrifuged at 5878 g for 10 min. Plasma concentrations of N-terminal pro-brain natriuretic peptide (NT-proBNP) and Troponin T were assayed with the Rat NT-proBNP ELISA kit (Cusabio Biotech Co., Ltd., Houston, TX, USA) and Rat cTnT/TNNT2 (Troponin T Type 2, Cardiac) CLIA Kit (#ER1396, Wuhan Fine Biotech Co., Ltd., Wuhan, China).

### 2.4. Examination of Cardiac Congestion and Lung Edema

After euthanizing the rats 6 h post-catecholamine infusion, hearts and lungs were removed and weighed. Heart-to-body weight ratio (%) was calculated by dividing heart weight by the body weight of the rats. The cranial, middle, and caudal lobe of the right lungs were weighed. Lung-to-body weight ratio (%) was calculated for lung edema index. Increased cardiac and lung-to-body weight ratio were used as surrogates of accumulated fluids and indicative of acute heart failure.

### 2.5. Histological Studies 

The lungs and heart were fixed with 10% formaldehyde and paraffin, then cut at a 4-μm slice thickness. Tissue sections were deparaffinized, rehydrated, then placed in either 10 mM sodium citrate buffer or Etope Retrieval Solution pH9 (#RE7119, Leica Biosystems Newcastle Ltd., Newcastle Upon Tyne, UK) at 90–100 °C for 20 min and were cooled to room temperature for heat-mediated antigen retrieval. Immunohistochemistry was visualized with the Novolink Polymer Detection Systems (Leica Biosystems Newcastle Ltd., Newcastle Upon Tyne, UK). Tissue sections were incubated in a peroxidase blocking solution (3–4% hydrogen peroxide) for 30 min, then treated with Protein Block for 30 min. Next, the sections were incubated sequentially with diluted rabbit primary antibodies and Anti-rabbit Poly-HRP-IgG reagent (Novolink Polymer). Primary polyclonal rabbit antibodies included the following: anti-connexin 43 (Cx43)/ GJA1 antibody (diluted 1:200 for heart and 1:400 for lungs, Abcam, #ab11370, Cambridge, UK), anti-fibroblast growth factor 23 (FGF-23) polyclonal antibody (Bioss Antibodies Inc., #bs-5768 R-TR, Wobum, MA, USA), anti-KL rabbit polyclonal antibody (1:50, Proteintech Group, Inc., #28100–1-AP, Rosemont, IL, USA), anti-high mobility group box protein 1 (HMGB1) rabbit polyclonal antibody (1:250, Proteintech Group, Inc., #10829–1-AP, Rosemont, IL, USA), anti-prosurfactant protein C (proSP-C) antibody (1:500, EMD Millipore Corporation, # AB3786, Temecula, CA, USA) and receptor for advanced glycation end products (RAGE) antibody (diluted 1:200, GeneTex Inc., Irvine, CA, USA). Endogenous production of reactive oxygen species of the cardiac tissue was observed via dihydroethidium (DHE) staining (Invitrogen). Deparaffinized tissue slices of 4 μm were subjected to 30 min staining of 1 μM DHE at 37 °C in a zero-light source area. The derived samples were analyzed and reviewed using a confocal microscope (Carl Zeiss LSM 5 PASCAL, Göttingen, Germany).

Finally, tissue sections were incubated in substrate/ chromogen, 3,3′-diaminobenzidine for 1 min, counterstained with hematoxylin and cover slipped. Results were photographed with a BX51 *p* polarizing microscope (Olympus Corp., Westborough, MA, USA). The sections were reviewed and graded by an experienced technician using a semiquantitative method, according to Liu et al. [12] and Jeschke et al. [13]. Immunohistochemistry was evaluated in the following two parts: sum of staining intensity (0 = no signal, 1 = weak, 2 = moderate, and 3 = strong) and proportion of positively stained cells within the 200-fold magnification field (0 ≤ 5% of cells, 1 = 5–25% of cells, 2 = 26–50% of cells, 3 = 51–75% of cells, and 4 = 76–100% of cells). The example sections used for reference were evaluated independently by another technician. The immunohistochemistry scores were calculated by summing up the staining intensity and proportion of positively stained cells.

### 2.6. Statistical Analysis

The data are expressed as mean ± SD, mean or median. All results were calculated using a nonparametric Kruskal-Wallis test, followed by a Mann-Whitney U-test. A *p* value of <0.05 was considered statistically significant. Statistical analyses were performed using IBM SPSS Statistics Version 20 software (IBM Corp., Armonk, NY, USA, 2011) and GraphPad Prism version 6.01 for Windows, (GraphPad Software. Inc., San Diego, CA, USA www.graphpad.com).

## 3. Results

### 3.1. Overall Survival, Organ-to-Body Weight Ratios and Serum Cardiac Biomarkers

**Figure 1:**
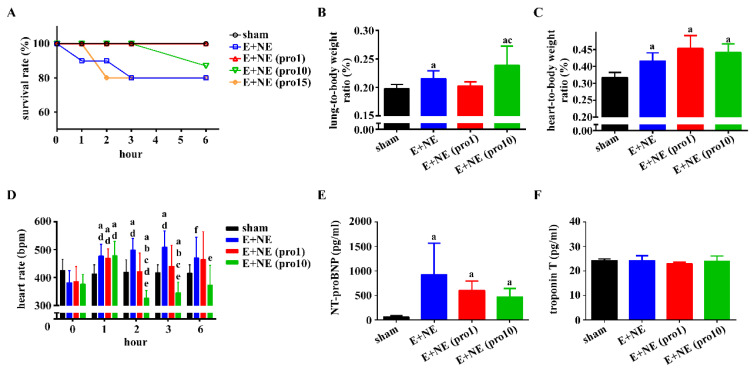
Low-dose propranolol improves overall survival and lung-to-body weight ratio. During catecholamine infusion alone or with additional propranolol over 6 h, the following data were measured. (**A**) Survival rate, sham, n = 6; E + NE, n = 10; E + NE (pro1), n = 7, E + NE (pro10), n = 8; E + NE (pro15), n = 10. (**B**,**C**) Organ-to-body weight ratio of the lungs and heart, respectively, in rats at the end of 6 h post-catecholamine or propranolol treatment. Sham, n = 5; E + NE, n = 6, E + NE (pro1), n = 7; E + NE (pro10), n = 7. (**D**) Heart rate, sham, n = 5; E + NE, n = 6; E + NE (pro1), n = 6; E + NE (pro10), n = 5. (**E**) Serum NT-proBNP changes, and (**F**) serum troponin T changes were measured in rat groups: Sham, n = 4; E + NE, n = 4; E + NE (pro1), n = 6; E + NE (pro10), n = 6. Data are represented as mean ± SD. Mann–Whitney U test was used for statistical analysis of all panels. ^a^ *p* < 0.05 vs. sham group; ^b^ *p* < 0.05 vs. E + NE group; ^c^ *p* <0.05 vs. E + NE (pro1) group; ^d^ *p* < 0.05 vs. respective group at 0 h; ^e^ *p* < 0.05 vs. respective group at 1 h; ^f^ *p* < 0.05 vs. respective group at 3 h. E + NE, epinephrine and norepinephrine; pro1, low-dose propranolol 1 mg/kg; pro10, high-dose propranolol 10 mg/kg; NT-proBNP, N-terminal pro-brain natriuretic peptide.

Hourly survival rates during catecholamine infusion were recorded for all subsets (Figure 1A). Low-dose propranolol (1 mg/kg) groups had the highest survival rates (100%), equal to the sham group. Groups with higher propranolol doses (15 mg/kg) had decreased survival rates, akin to E and NE only groups. This is consistent with working groups studying propranolol overdose in rat models [14]. Trials of greater propranolol doses (20, 25 mg/kg) were administered, however due to 100% mortality, only low-dose and high-dose propranolol were selected for therapeutic investigation. Lung-to-body weight ratios increased significantly in combined catecholamine and high-dose propranolol subsets, with low-dose propranolol groups having similar ratios to the sham groups (Figure 1B). Heart-to-body weight ratios were not attenuated with propranolol, regardless of dosage (Figure 1C). Heart rates of low-dose propranolol groups were similar to the sham groups at hour 2, 3, and 6. Only the high-dose propranolol subset exhibited decreased heart rates at hour 2 and 3 (Figure 1D). Serum NT-proBNP was significantly increased in all three experimental groups, compared to sham groups (Figure 1E). No significant change in serum troponin T was noted among all groups (Figure 1E).

### 3.2. Hemodynamic Changes of the LV

**Figure 2:**
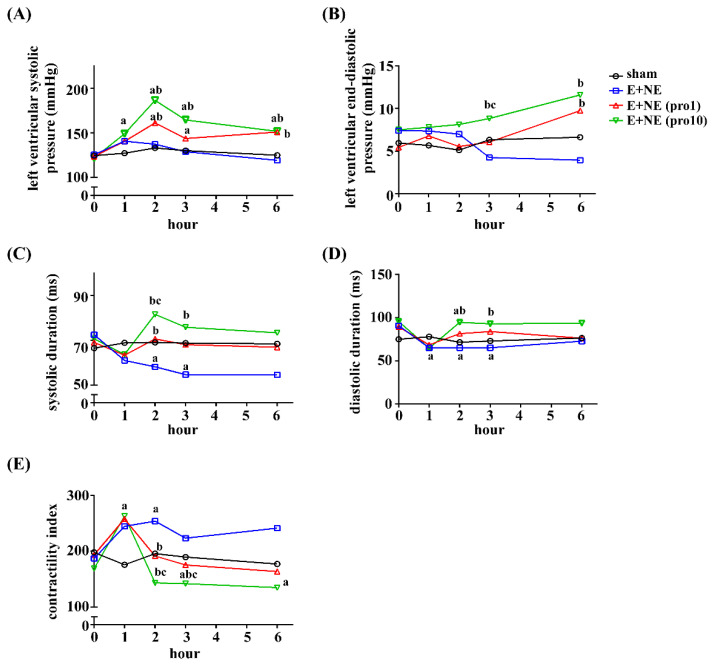
Trend graphs of hemodynamic changes of the left ventricle in rat models with excessive catecholamine infusion alone or with additional propranolol treatment. Measurements of (**A**) left ventricular systolic pressure, (**B**) left ventricular end-diastolic pressure, (**C**) systolic duration, and (**D**) diastolic durations were recorded. These measurements were assessed in the following rat groups: Sham, n = 5; E + NE, n = 6; E + NE (pro1), n = 7; E + NE (pro10), n = 6. (**E**) Contractility indexes were measured in the following rat groups: Sham, n = 5; E + NE, n = 6; E + NE (pro1), n = 6; E + NE (pro10), n = 5. Data are represented as median. Mann–Whitney U test was used for statistical analysis of all panels. ^a^ *p* < 0.05 vs. sham group; ^b^ *p* < 0.05 vs. E + NE group; ^c^ *p* < 0.05 vs. E + NE (pro1) group. E + NE, epinephrine and norepinephrine; pro1, low-dose propranolol 1 mg/kg; pro10, high-dose propranolol 10 mg/kg.

Left ventricular systolic pressure (LVSP) remained significantly elevated after hour 2 in high- and low-dose propranolol groups, when compared to the sham and catecholamine-only groups (Figure 2A). The left ventricular end-diastolic pressure (LVEDP) of high-dose propranolol groups also significantly increased when compared to catecholamine-only groups at hour 3 and 6, as well as low-dose propranolol groups at hour 3 (Figure 2B). In catecholamine-only groups, systolic durations of the LV decreased at hour 2 (sham, 0.07 ± 0.005 ms, E and NE, 0.06 ± 0.006 ms, *p* < 0.05) and hour 3 (sham, 0.07 ± 0.004 ms, E and NE, 0.06 ± 0.007 ms, *p* < 0.05). Diastolic durations of the LV decreased at hour 2 (sham, 0.08 ± 0.013 ms, E and NE, 0.06 ± 0.004 ms, *p* < 0.05) and hour 3 (sham, 0.08 ± 0.008 ms, E and NE, 0.07 ± 0.005 ms, *p* < 0.05). After low-dose propranolol treatment, systolic durations were similar to the sham groups at hour 2, 3 and 6 (respectively, sham, 0.07 ± 0.005 ms, low-dose, 0.07 ± 0.009 ms, *p* > 0.05; sham, 0.07 ± 0.004 ms, low-dose, 0.07 ± 0.011 ms, *p* > 0.05; sham, 0.07 ± 0.004 ms, low-dose, 0.06 ± 0.012 ms, *p* > 0.05). In high-dose propranolol groups, systolic and diastolic durations were significantly prolonged (*p* < 0.05, compared to the E and NE group at hour 2 and 3, Figure 2C,D). The contractility indexes of all groups peaked at hour 1, with a significant dose-dependent decline in the treatment groups (Figure 2E).

### 3.3. Hemodynamic Changes of the RV

**Figure 3:**
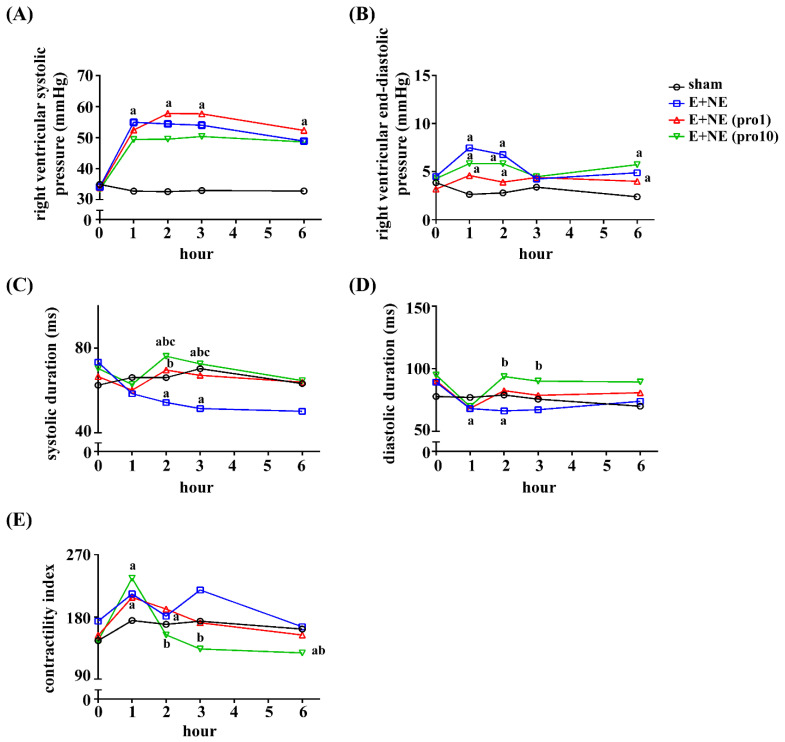
Trend graphs of the hemodynamic changes of the right ventricle in rat models with excessive catecholamine infusion alone or with additional propranolol treatment. (**A**) Right ventricular systolic pressure, and (**B**) right ventricular end-diastolic pressure, were recorded in rat groups of sham, n = 5; E + NE, n = 6; E + NE (pro1), n = 7; E + NE (pro10), n = 6. (**C**) Systolic durations were measured in groups of sham, n = 5; E + NE, n = 6; E + NE (pro1), n = 7; E + NE (pro10), n = 5. (**D**) Diastolic durations were measured in groups of sham, n = 5; E + NE, n = 6; E + NE (pro1), n = 7; E + NE (pro10), n = 6. (**E**) Contractility indexes were measured in groups of n = 5 for all conditions, except for E + NE (pro10), n = 6. Data were presented as median. Mann–Whitney U test was used for statistical analysis of all panels. ^a^ *p* < 0.05 vs. sham group; ^b^ *p* < 0.05 vs. E + NE group; ^c^ *p* < 0.05 vs. E + NE (pro1) group. E + NE, epinephrine and norepinephrine; pro1, low-dose propranolol 1 mg/kg; pro10, high-dose propranolol 10 mg/kg.

In the E and NE group, the immediate elevation of the right ventricular systolic pressure (RVSP) and right ventricular end-diastolic pressure (RVEDP) (Figure 3A,B) was noted after the administration of catecholamines. A lack of improvement in RVSP and RVEDP was observed in the low-dose and high-dose propranolol groups (hour 1 through 6, compared to E and NE, *p* > 0.05 in low-dose and high-dose groups, Figure 3A,B). Shortened systolic durations were observed at hour 2 (sham, 0.07 ± 0.005 ms, E and NE, 0.05 ± 0.007 ms, *p* < 0.05) and hour 3 (sham, 0.07 ± 0.004 ms, E and NE, 0.05 ± 0.007 ms, *p* < 0.05) (Figure 3C,D). Shortened diastolic durations were also noted (sham, 0.08 ± 0.009 ms, E and NE, 0.07 ± 0.007 ms, *p* < 0.05) (Figure 3C,D). At hour 2, 3, and 6 after low-dose propranolol treatment, normalized systolic duration (sham, 0.07 ± 0.005, low-dose, 0.06 ± 0.013 ms, *p* > 0.05) (Figure 3C) and normalized diastolic duration (sham, 0.07 ± 0.010 ms, low-dose, 0.08 ± 0.015 ms, *p* > 0.05) (Figure 3D) were observed. 

The administration of high-dose propranolol caused a marked prolongation of both systolic and diastolic durations, observed at hour 2, 3 and 6 (*p* < 0.05, compared with E and NE, Figure 3C,D). The contractility index changes of the RV were similar to that of LV, with maximal contractility indexes observed at 1 h post-catecholamine administration. A subsequent proportional decrease in the contractility index with low-dose and high-dose propranolol was observed (Figure 3E). 

### 3.4. Acute Cardiac Injury in Histopathology

**Figure 4:**
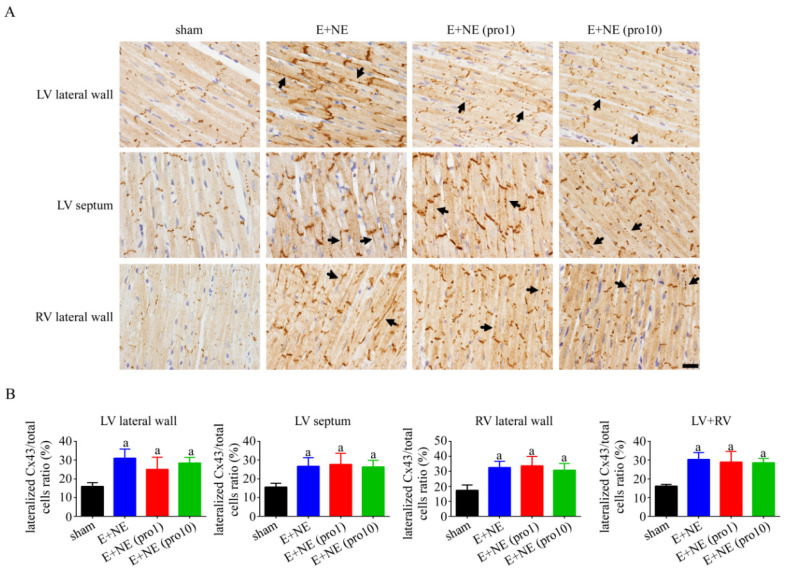
Changes in connexin 43 distribution reveal lateralization in cardiac tissue with 6 h continuous catecholamine infusion alone or with propranolol treatment. (**A**) Representative immunohistochemical staining with connexin 43 antibodies in cardiac tissue and (**B**) quantitative analysis of lateralized Cx43 in LV lateral wall, LV septum, and RV lateral wall of after 6 h in rat groups of: sham, n = 4; E + NE, n = 5; E + NE (pro1), n = 5; E + NE (pro10), n = 5. Data are represented as mean ± SD. Mann–Whitney U test was used for statistical analysis of all panels. ^a^ *p* < 0.05 vs. sham group. Cx43, connexin 43; E + NE, epinephrine and norepinephrine; LV, left ventricle; pro1, low-dose propranolol 1 mg/kg; pro10, high-dose propranolol 10 mg/kg; RV, right ventricle. The scale bars represent 50 μm.

**Figure 5:**
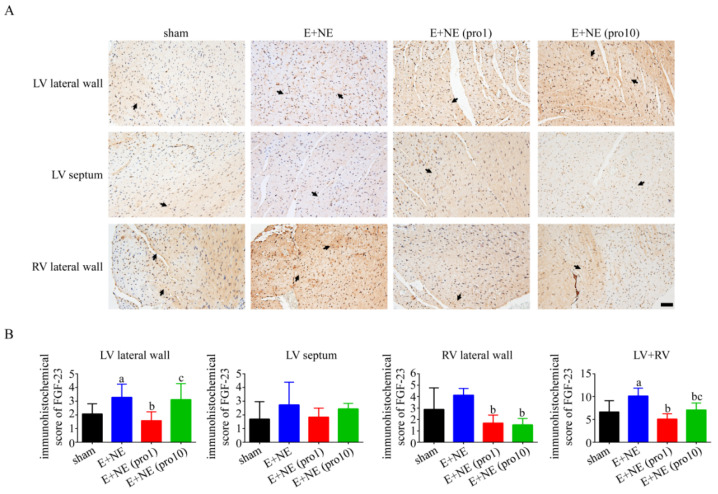
Expression of fibroblast growth factor-23 (FGF-23) in cardiac tissue after 6 h infusion of catecholamine alone or with propranolol treatment. (**A**) Representative immunohistochemical staining with FGF-23 antibodies, and (**B**) the immunohistochemical scores of FGF-23 in LV lateral wall, LV septum, and RV lateral wall after 6 h in groups of: sham, n = 6; E + NE, n = 6; E + NE (pro1), n = 6; E + NE (pro10), n = 5. Data are expressed as mean ± SD. Mann–Whitney U test was used for statistical analysis of all panels. ^a^ *p* < 0.05 vs. sham group; ^b^ *p* < 0.05 vs. E + NE group; ^c^ *p* < 0.05 vs. E + NE (pro1) group. E + NE, epinephrine and norepinephrine; LV, left ventricle; pro1, low-dose propranolol 1 mg/kg; pro10, high-dose propranolol 10 mg/kg; FGF-23, fibroblast growth factor-23; RV, right ventricle. The scale bars represent 50 μm.

Cardiac injury was investigated with the immunohistochemistry of connexin 43 (Cx43) for lateralization in cardiomyocytes [15]. Lateralization of Cx43 in the LV lateral wall, ventricular septum, and RV lateral wall was observed after catecholamine infusion, without significant changes in the high-dose and low-dose propranolol treatments (see Figure 4). FGF-23 upregulation was observed after continuous catecholamine infusion. The attenuation of FGF-23 in the LV wall was observed after propranolol administration. Maximal FGF-23 downregulation to near-normalization was observed in the low-dose propranolol groups (Figure 5). An examination of the klotho protein and reactive oxygen species immunohistochemistry denied significant differences among all groups (see Supplementary Figures S1 and S2, respectively).

### 3.5. Lung Injury Mediated through Apoptosis

**Figure 6:**
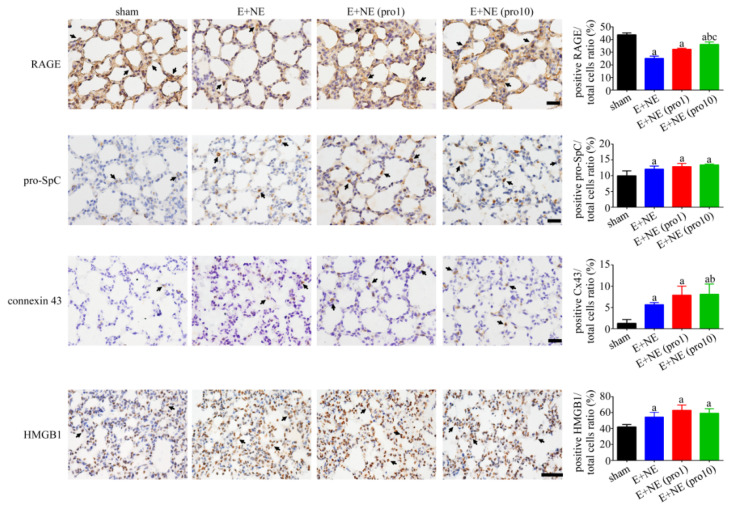
Differences in protein expression among groups in lung tissue after catecholamine-induced toxicity alone or with propranolol treatment. Representative immunohistochemical staining and quantitative analysis of RAGE, pro-SpC, Cx43, and HMGB1 in lung tissues after 6 h in sham, E + NE, E + NE (pro1), and E + NE (pro10) groups. n = 4 in all groups except for the sham group in quantitative analysis of Cx43, n = 3. Data are expressed as mean± SD. Mann–Whitney U test was used for statistical analysis of all panels. ^a^ *p* < 0.05 vs. sham group; ^b^ *p* < 0.05 vs. E + NE group; ^c^ *p* < 0.05 vs. E + NE (pro1) group. Cx43, connexin 43; E + NE, epinephrine and norepinephrine; HMGB1, High mobility group box 1; pro1, low-dose propranolol 1 mg/kg; pro10, high-dose propranolol 10 mg/kg; pro-SpC, pro-surfactant protein C; RAGE, receptor for advanced glycation end products. The scale bars represent 50 μm.

In pulmonary tissue, a significantly downregulated RAGE and an upregulation of pro-surfactant protein C, Cx43, and HMGB-1 expression were observed 6 h post catecholamine infusion. Low-dose and high-dose propranolol subsets failed to modify the expression of pro-surfactant protein C, Cx43, and HMGB-1. However, RAGE was upregulated after high-dose propranolol treatment (Figure 6).

## 4. Discussion

Our rat models of combined catecholamine-induced heart failure, observed over 6 h, demonstrated the prevention of cardiac dysfunction with a low-dose of bolus propranolol. To the best of our knowledge, no previous animal studies have attempted to realistically simulate hyperadrenergic states through combined catecholamines. Furthermore, a low-dose propranolol bolus effectively mitigated acute heart failure and prolonged survival. Finally, pathological changes of early cardiac dysfunction may be mediated through FGF-23-dependent mechanisms, with maximal prevention after the administration of low-dose propranolol. Our experiment demonstrates that the deleterious effects of adrenergic stimulation are best prevented through low-dose propranolol (1 mg/kg). Therefore, we propose a catecholamine-induced heart failure model, with the prevention of cardiac dysfunction mediated through low-doses of beta-blockade.

Few studies have reported animal models of combined catecholamine-induced cardiomyopathy. Previous animal models were mostly chronically exposed to either E or NE, leading to ventricular dysfunction [16,17,18]. Chronic high-dose E infusion (7.5 mg/kg/min) in rats induced biventricular ischemia and fibrosis, while continuous NE (0.1 mg/kg/h) infusion induced LV hypertrophy and fibrosis [19]. Yet, despite the plethora of animal models utilizing individual catecholamines for heart failure models, a combination of E and NE has not been broadly adopted for acute heart failure models. However, we believe this catecholamine-induced heart failure model is also compatible with clinical scenarios of hyper-catecholaminergic states, including sepsis [1], chronic heart failure [20], and catecholamine administration during resuscitation [21]. 

In our preceding study, we investigated the physiological changes of excessive catecholamine infusions in rat models with parameters such as echocardiography and ventricular pressure. Infusions of high-dose E (4.5 µg/kg/min) and NE (6.8 µg/kg/min) over 6 h in rats were observed to cause a thickened interventricular septum and increased left ventricular mass upon echocardiography, reflecting cardiomyocyte hypertrophy. A significantly reduced stroke volume, cardiac output, systolic and diastolic function were all found in combined E and NE infusion groups as well, signifying cardiac dysfunction [10]. 

Of the hemodynamic parameters measured in our current study, the catecholamine-only subsets reflected similar declining LVSP and LVEDP changes as in our previous study groups, reflecting adverse cardiac hemodynamic changes through initially high cardiac output states with subsequent cardiac dysfunction. After low-dose propranolol treatment, LVSP, LVEDP and the contractility indexes improved significantly, preventing cardiac dysfunction through cardiac function optimization. This is consistent with propranolol’s beta-1 antagonism properties, maintaining cardiac output and decreasing the myocardial oxygen demand through decreased heart rate, longer diastolic duration and higher end-diastolic volume. These results provide mechanistic explanations for recent studies investigating the benefits of optimized heart rates and hemodynamics with beta-blockers in hyper-catecholaminergic states [22]. In high-dose propranolol groups, the high mortality rate is likely due to its strong membrane action, potential stabilization properties and over-suppression of the adrenergic system as elevated LVEDP was observed [23]. It is worthwhile to note that at even higher doses of propranolol treatment (15 mg/kg), mortality was higher than the high-dose group and on par with the E-and-NE group. Previous lethal dosing of propranolol in rats was documented at 15 mL/kg, with cause of death due to clinical suppression of the central neurological system through membrane action potential stabilization, and direct cardiac depressant effects such as PR prolongation and AV dissociations [14,24]. However, the rats in previous studies were not pre-treated with combined catecholamines, therefore, rat groups with sequential boluses of combined catecholamines and 15 mg/kg propranolol were included in the initial stage of our study. Further analyses of this group were not performed as their mortality rate was similar to the combined-catecholamine rat group. 

The hemodynamic trends of RV were slightly different from those of LV. The catecholamine-alone group had an overall slower right ventricular response than the LV, likely due to ventricular anatomical and physiological differences. The RV has less muscle mass with thinner walls and is therefore acutely sensitive to afterload changes leading to dysfunction [25]. Another reason for different ventricular responses may be the heterogenous distribution of adrenergic receptors between the ventricles; however, previous rat studies suggest that the distribution of beta-adrenoreceptors is not significantly different among ventricles [26]. 

We further investigated the histopathological changes of propranolol upon the myocardium after catecholamine injury. It was previously observed that with supratherapeutic doses of NE and E, the lateralization of Cx43 through reactive oxidative stress pathways occurs in response to severe stress [10]. In vitro studies of cultured rat cardiomyocytes exposed for 24 h to norepinephrine (1–10,000 nM) also increased their expression of Cx43, while in vivo equivalents showed an association of catecholamine stimulation with enhanced gap junctional intercellular communication [27]. Our results are consistent with other working groups that used isoproterenol, a β1- and β2 -adrenoceptor agonist, as a medium to stimulate neonatal cardiomyocytes, with findings of upregulated Cx43 protein in both isoproterenol-only and combined isoproterenol-metoprolol mediums [28]. The lateralization of Cx43 was not improved by either of the propranolol groups within our 6 h observation period, implicating clinical-histopathological incompatibility with early signs of pathological acute cardiac injury despite perceived clinical hemodynamic reversal. To the best of our knowledge, the role of catecholamines and possible treatment effects of beta-blockers upon Cx43 expression has not been well-researched in the literature, and our pathological findings suggest that acute cardiac injury and subsequent pathological compensatory mechanisms are persistent despite propranolol treatment within six hours. 

Next, we investigated possible participating molecular pathways in acute hemodynamic changes after adrenergic overstimulation through the staining of FGF-23. Faul et al. demonstrated pathological left ventricular hypertrophy through the direct intraventricular injection of FGF-23 [29], likely through klotho-independent pathways [30]. While there are known associations of elevated FGF-23 and cardiovascular morbidities in chronic kidney diseases in both clinical and experimental studies, less is understood in an acute, hyperadrenergic setting [31,32]. Previous investigations associated FGF-23 with causal myocardial fibrosis during acute cardiac injury [33]. In our study, FGF-23 was significantly upregulated in the LV, and though upregulated in the RV, failed to reach significance. This suggests an early initiation of the pro-fibrotic pathway in bilateral ventricles through FGF-23 upregulation in hyper-catecholaminergic states. It is also worthy of note that an increased expression of FGF-23 in the RV was not previously observed in intramyocardial injections of FGF-23 [29]. 

Low-dose propranolol was the only treatment group to effectively downregulate FGF-23 in both ventricles, likely due to optimal cardiac stabilization as evidenced by hemodynamic parameters, with high-dose propranolol only effective in the RV. FGF-23 reversal is thus corroborative of the compatibility between ventricular function and histological changes, further evident of the pathological prevention of adverse cardiac dysfunction. FGF-23 upregulation was only significant in the LV lateral wall of the high-dose propranolol group, likely secondary to the overt adrenergic suppression of the LV, leading to volume overload with acute decompensated heart failure. 

Our present catecholamine-induced heart failure model, while similar to various hyper-adrenergic states, may not be strictly compatible with specific clinical scenarios due to their variability. Another potential limitation is the lack of pressure-volume loop analyses, meaning that detailed measurements such as ejection fraction were not collected. Furthermore, immunohistochemistry could be refined with the identification of beta-selective receptors, and downhill kinases could be included for an investigation of different pathways. In addition, we were unable to exclude the role of inflammation in catecholaminergic acute lung injury due to a lack of relevant markers, and the experiment time of 6 h may not have been long enough for the development of lung apoptosis. Finally, we were unable to determine the precedence of cardiac and pulmonary injury as the accruement of murine tissues was only possible at the end of the 6 h experiment, instead of detailed time points at hour 1 through 6. While cardiac function improved with propranolol administration, the negative findings in histopathological markers including HMGB-1, Cx-43 and prosurfactant apoprotein-C may be limited by the short 6 h experiment period; therefore, the compatibility of clinical and histopathological changes is still unclear. 

## 5. Conclusions

In conclusion, this experiment demonstrated that a single low-dose propranolol of 1 mg/kg was successful in improving survival and hemodynamics in a combined catecholamine-induced acute heart failure model. The therapeutic effects of propranolol were not dose-dependent, as high-dose and higher-dose propranolol both caused lower survival rates. Myocardial FGF-23 expression, a pro-fibrotic marker, was also downregulated with propranolol treatment, implicating the role of non-selective beta blockers in the prevention of cardiac dysfunction. Low-dose propranolol may therefore be useful as a cardioprotective treatment option in the hyper-acute setting of excess catecholaminergic states with acute heart failure. 

## Data Availability

Not applicable.

## Supplementary Materials

The following supporting information can be downloaded at: https://www.mdpi.com/article/10.3390/toxics10050238/s1, Figure S1: Klotho expression in the heart tissue 6 h post continuous combined catecholamine infusion alone, or with propranolol treatment, Figure S2: Level of reactive oxygen species in heart tissues 6 h post excess catecholamine infusion alone, or with propranolol treatment.
