# Supplementary material for: Low-Dose Propranolol Prevents Functional Decline in Catecholamine-Induced Acute Heart Failure in Rats
Source: Toxics. 2022 May 7;10(5):238. doi: 10.3390/toxics10050238 (PMC9148026; doi:10.3390/toxics10050238)
Supplement: Supplementary file 1 [file toxics-10-00238-s001.zip › toxics-1699397-supplementary.pdf]

toxics

MDPI

Supplementary Materials: Low-dose Propranolol Prevents Functional Decline in Catecholamine-Induced Acute Heart Failure in Rats

Cheng-Ken Tsai, Bo-Hau Chen, Hsin-Hung Chen, Rebecca Jen-Ling Hsieh, Jui-Chen Lee, Yi-Ting Chu and Wen-Hsien Lu

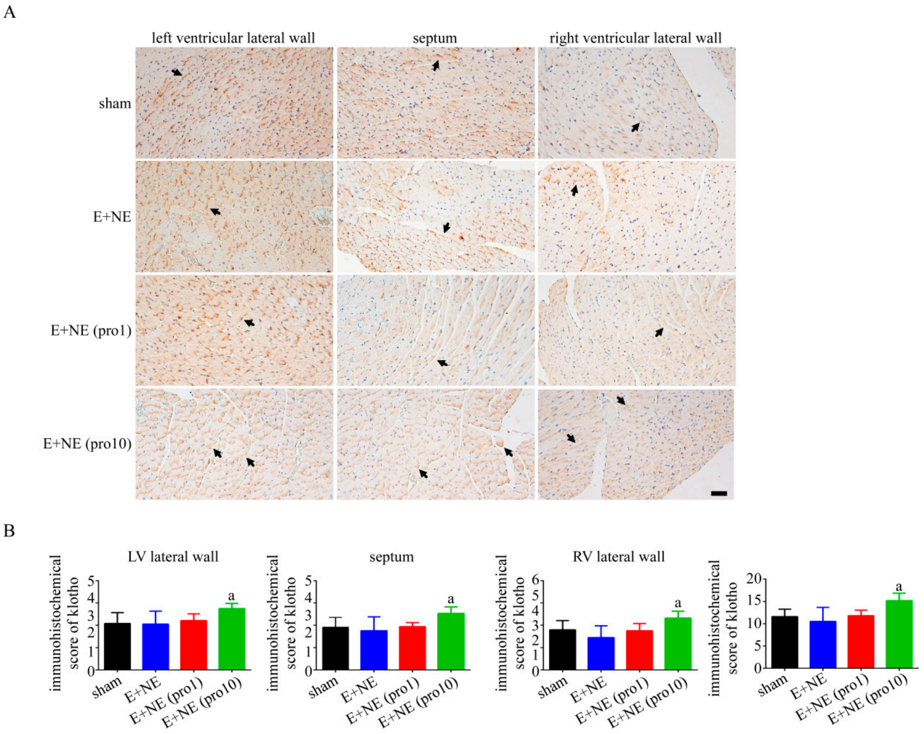

Figure S1. Klotho expression in the heart tissue 6 hours post continuous combined catecholamine infusion alone, or with propranolol treatment. (**A**) Representative immunohistochemical staining with klotho antibodies in cardiac tissue, and (**B**) immunohistochemical scores of klotho in LV lateral wall, LV septum, and RV lateral wall of the following groups: sham, n = 8; E+NE, n = 8; E+NE (pro1), n = 6; E+NE (pro10), n = 5. Data are expressed as mean ± SD. Man-Whitney U test was used for statistical analysis of all panels. [a]$p < 0.05$ vs. sham group. E+NE, epinephrine and norepinephrine; LV, left ventricle; pro1, low-dose propranolol 1 mg/kg; pro10, high-dose propranolol 10 mg/kg; RV, right ventricle. Scale bar = 50 um.

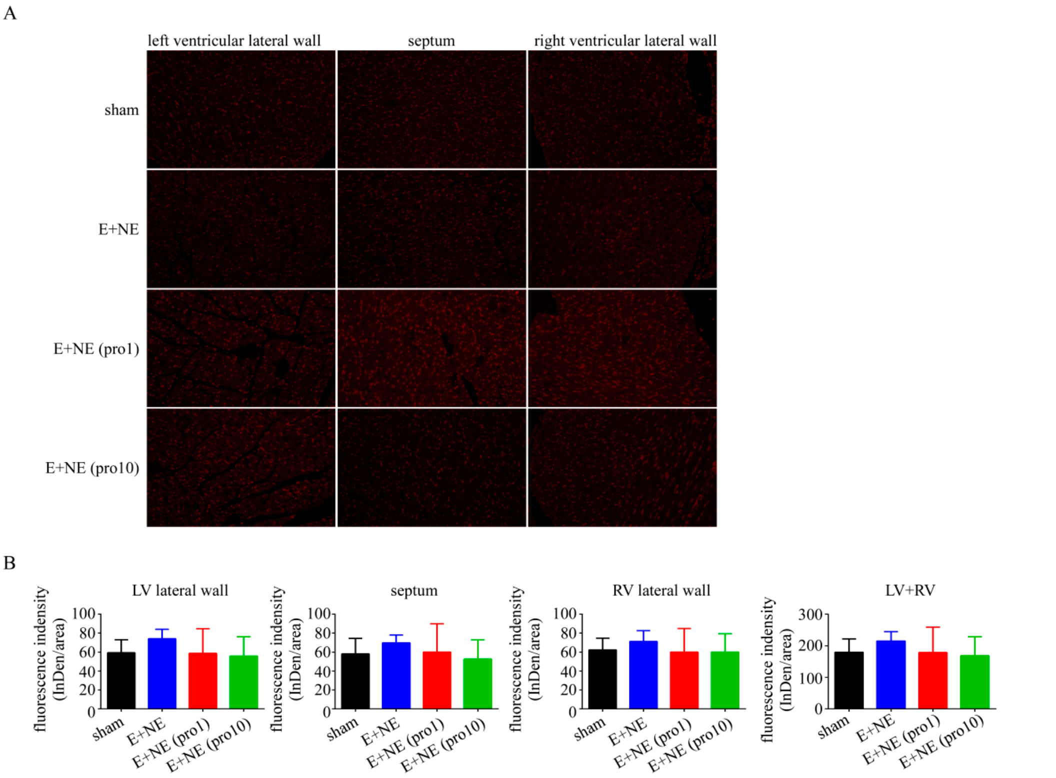

Figure S2. Level of reactive oxygen species in heart tissues 6 hours post excess catecholamine infusion alone, or with propranolol treatment. (**A**) Representative immunofluorescent images of dihydroethidium staining, and (**B**) quantitative analysis in LV lateral wall, LV septum, and RV lateral wall in groups of sham, n = 4; E+NE, n = 4; E+NE (pro1), n = 3; E+NE (pro10), n = 3. Data are expressed as mean ± SD. Man-Whitney U test was used for statistical analysis of all panels. E+NE, epinephrine and norepinephrine; LV, left ventricle; pro1, low-dose propranolol 1 mg/kg; pro10, high-dose propranolol 10 mg/kg; RV, right ventricle.
